# The effect of cardiac phase on distractor suppression and motor inhibition in a stop-signal task

**DOI:** 10.1038/s41598-024-80742-2

**Published:** 2024-12-02

**Authors:** Amanda C. Marshall, Qiaoyue Ren, Lioba Enk, Junhui Liu, Simone Schütz-Bosbach

**Affiliations:** 1 Department of Psychology, General and Experimental Psychology Unit, LMU Munich, Leopoldstr. 13, D-80802, Munich, Germany; 2 Max-Planck-Institute for Human Cognitive and Brain Sciences, Stephanstr. 1a, 04103 Leipzig, Germany; 3 Max Planck School of Cognition, Stephanstr. 1a, 04103 Leipzig, Germany

**Keywords:** Cardiac cycle, Distractor processing, EEG, Interoception, Motor inhibition, Psychology, Human behaviour

## Abstract

Past work has shown that stimuli timed to the cardiac systole, the time at which heartbeat feedback is transmitted to the brain, can be more effectively selected from conflicting information. Here, we investigated how the temporal alignment of distracting information to different phases of the cardiac cycle impacts inhibitory performance on a stop-signal task. While participants received the go-cue and anticipated a potential stop-signal, we presented several moving dots on the screen. The dots’ change of movement direction was timed to occur 290 ms posterior to the R-peak (for cardiac systole) or right at the R-peak (for cardiac diastole) of in-time ECG recordings. In a third control condition, no distracting dots were shown. Behavioural results found participants were significantly better at inhibiting their motor response in systole relative to diastole distractor trials. Electrophysiological evidence found reduced P2 amplitudes for viewing the distractors and enhanced N2 amplitudes to the subsequent stop-signal in systole relative to diastole distractor trials. This indicated that systole bound distractors were suppressed more effectively than diastole bound ones which led to enhanced motor inhibition. Our results indicate that the brain shows greater visual selection efficiency for distracting information co-occurring with cardiac systole which has implications for enhanced motor processing at later stages of the trial sequence.

**Supplementary Information:**

The online version contains supplementary material available at 10.1038/s41598-024-80742-2.

## Introduction

Our perception and cognition, our experience of the world at large, is strongly influenced by bodily states. As such, a growing body of empirical work investigates the way fluctuating internal rhythms such as brain oscillations, heartbeats or breath cycles are processed and integrated with external sensory information to enable a dynamic and adaptive interaction with the environment^1,2^. The heartbeat, as one of the body’s most prominent interoceptive signals conveyed to the brain, has received much attention in this regard with studies investigating how different phases of the cardiac cycle relate to the uptake and processing of external information^3,4^. The cardiac cycle consists of two phases. During diastole, the heart’s ventricles relax and refill with blood. At systole, the ventricles contract, sending blood into the arteries. Contraction at systole is picked up by baroreceptors in the arterial and carotid arteries which convey information about the timing and strength of heartbeats to cortical areas such as the insula. At diastole, those receptors fire only intermittently, the brain thus receives strong and consistent heartbeat feedback only at systole. Several studies have utilised this phenomenon, pairing stimuli with different phases of the cardiac cycle to explore how the processing of external sensory input is affected by information arriving in conjunction with or distinct from cortical heartbeat feedback. However, it is important to note, that while cardiac phase effects on cognition have been linked to baroreceptor firing, causal evidence for this link is still lacking. In addition, the exact way in which experimental performance has been linked to the cardiac cycle differs across studies: some have time locked stimulus onsets or behavioural commands to exact time points within the ongoing ECG stream, for instance to the approximated arrival of baroreceptor activity to the brain (around 300 ms posterior to the preceding R-peak); others combine ECG recordings and jittered trial designs to then a posteriori link experimental events to cardiac phases (such as the systole or diastole time window) after the data has been collected. It is important to note that the first design, which we have also adopted in the current study, may bring about temporal predictability of contingencies between experimental and cardiac dynamics while the latter approach will not. Based on these issues, we would like to highlight that mechanistic caution should be applied when interpreting the underlying cause of cardiac cycle effects. In addition, the current methodological discrepancies emphasize the importance for a clear notation with regards to the method by which the cardiac cycle is related to external stimulus intake and future work would benefit from a standardized definition of systole and diastole coupling to enhance comparability across future studies.

In the visual domain, past work has used the a posteriori approach to place events in different windows of the cardiac cycle. Results highlight that features of visual information sampling, such as microsaccades and drifts in fixational eye movements, are locked to the cardiac cycle^5^. For example, Galvez-Pol, McConnell and Kilner^6^ recorded the number of fixations, saccades and blinks which fell into different points of the cardiac cycle while participants evaluated the differences between two visual arrays. They observed that longer fixations (i.e. intake of visual information) occurred during the diastolic time window while participants performed more saccades during the systolic time window. Conversely, past work has also shown that the intake of visual information is voluntarily timed by participants to occur in conjunction with the cardiac systole. To this effect, Kunzendorf and colleagues^7^ gave participants a memory task in which they were allowed to self-initiate the onset of a shortly presented image they had to memorise. Likewise, the authors observed that most display onsets were timed by participants to occur within the time window of their cardiac systole. Through time-locking stimulus onsets to specific time points in the ECG, Garfinkel and colleagues^8^ demonstrated that fearful faces shown at the threshold of conscious detection were identified more efficiently and rated as more intense when presented at systole (R-peak + 300 ms). Complementing the picture further, Salomon and colleagues^9^ reported reduced detection accuracy in a visual masking experiment when the target stimulus was coupled to cardiac diastole (R-peak + 0), relative to being presented asynchronously to the heartbeat, while Pramme and colleagues^10,11^ further demonstrated that visual interference of a masking stimulus could be more readily inhibited when target and mask were presented simultaneously at systole (R-peak + 290 ms). Similarly, our own work^12^ has shown that synchronising external stimuli to the cardiac systole (R-peak + 290 ms) leads to enhanced repetition-suppression of the subsequent exteroceptive stimulus. Past work hereby denotes that the relative timing of visual input within the cardiac cycle does not only affect the selection efficiency of such stimuli but may also be reflected in the inhibition of competing visual information, suggesting that time-locking external input to baroreceptor feedback may act as a noise cancellation mechanism resulting in decreased perception of weak sensory input while simultaneously increasing the perception of salient exteroceptive information.

Within the motor domain, the impact of cardiac phase on action is widely known. For example, biathletes are trained to shoot in the interval of consecutive heartbeats to counteract the effect of their accelerated heartrate on the fine motor control necessary for hitting the target^13^. Further studies in this domain have focused on motor inhibition. However, empirical work in this regard remains scarce and provides conflicting findings. Rae and colleagues^14,15^, for example, investigated the effect of cardiac phase on inhibitory motor performance by timing stimuli to exact timepoints of the ECG across two tasks. In a modified Go/NoGo task participants were presented with go, no go, and choice reaction targets for which they could decide whether to provide or withhold a motor response. Target appearance was linked to either cardiac systole (R-peak + 290 ms) or diastole (R-peak − 50 ms). In this instance, the authors did not find an effect of cardiac phase on intentional motor inhibition. However, a significant effect of cardiac phase emerged in a stop-signal task in which the appearance of the stop cue, the colour change of an arrow coupled to a tone, was linked either to the cardiac systole (R-peak + 290) or diastole (R-peak – 50 ms). The authors observed that response inhibition significantly improved when the stop cue co-occurred with cardiac systole. Yet, work from our lab^16^ observed the opposite pattern. For a stop-signal task in which the stop signal was provided either in the form of a colour change or an emotional expression, we observed prolonged stop-signal reaction times in systole (R-peak + 290 ms) coupled compared to uncoupled (i.e., randomly presented) stop trials. In addition, electrophysiological indices of inhibitory motor and cortical heartbeat processing suggested a trade-off mechanism between internal and external cognitive resources: amplitudes of the stop-signal P3 were lower at systole while amplitudes of the Heartbeat Evoked Potential (HEP), a cortical marker of heartbeat processing, were higher. We observed a similar trade-off in an earlier contribution where we report a negative correlation between the HEP and the amplitude of the feedback P3 ERP component in response to accuracy feedback in a reward incentive motor task^17^. Our findings here suggested that the efficiency of interoceptive processing may also impact the efficacy with which we process feedback cues to guide subsequent motor behaviour.

Current research thus seems to propose a putative effect of afferent cardiac signalling on stimulus processing. However, baroreceptor influence on external perception co-exists and interrelates with gastric and respiratory modulations of informational intake^18–20^ and is modulated by stimulus characteristics and individual traits such as interoceptive sensitivity^21^. Moreover, baroreceptor signalling at systole coincides with several physiological fluctuations which may affect the way stimuli are perceived^22–24^ as well as the efficacy with which baroreceptors transmit cardiac activity to relevant hubs in the brain^25,26^. Thus, it is at this point, unclear whether the observed effects in the literature are due to the exclusive effects of cardiac baroreceptor signalling.

Our study aims to shed further light on the effect of cardiac cycle timing on motor inhibition and motor feedback processing. Work to date has not yielded consensus whether the effects of cardiac phase on inhibitory motor performance arise from a potential trade-off between internal and external processing resources or from a general facilitation or debilitation of external signal processing by linking them to interoceptive cues. To explore these conflicting interpretations, we combined foregone research in the visual and motor domain to test whether the presence of distracting information linked to the cardiac cycle impacts inhibitory performance. We used a modified stop-signal task in which a stop-signal succeeded a go-cue in a third of all trials. While being presented with the go cue and potentially a stop-signal, participants were tasked to ignore a set of moving dots. The onset of the dots’ directional change of movement was timed to occur at an exact timepoint of the recorded ECG signal either within the participants’ cardiac systole (R-peak + 290 ms) or diastole (R-peak + 0 ms). To ensure that participants’ attentional focus included the distractors, we chose a globally presented stop-signal^27^ in the form of a border lining the edges of the screen. To explore the mechanisms underlying the effect of cardiac phase on cognitive processes, we paired our paradigm with electrophysiological recordings. We chose the HEP as an electrophysiological index of interoceptive heartbeat processing, as well as event-related potentials reflecting visual, inhibitory motor and error feedback processing. Based on past work indicating greater visual selection efficiency when conflicting information is presented at cardiac systole^10,11^ we hypothesised reduced distractor interference for trials in which distractor activity co-occurs with the cardiac systole. We expected this to produce enhanced behavioural performance on the stop-signal task. We further anticipated distinct patterns of electrophysiological activity reflecting the interplay of external vs. internal processing. In line with Ren and colleagues^16^, we expected enhanced HEP amplitudes, coupled with reduced visual evoked potential (VEP) amplitudes evoked by systole coupled distractors, thus reflecting the successful inhibition of distracting information. We further hypothesised that successful distractor inhibition for systole trials would result in enhanced inhibitory motor indices as well as increased activity related to error feedback processing. For diastole trials in which distractor movement and heartbeat feedback are displaced, we expected reduced HEP amplitudes, coupled with higher VEP activity reflecting greater cortical processing of distracting information. We further expected that this would lead to reduced indices of motor inhibition and feedback processing as well as to reduced behavioural performance on the stop-signal task.

## Method

### Participants

Forty participants (18 females, 0 diverse, 22 males; years of age: 25.3 ± 3.9 y [mean ± SD; range: 19 to 33 y]) with no reported history of psychiatric or neurological disorders and no intake of medications affecting neural or physiological function took part in the study. Required sample size was determined before data acquisition. A power analysis indicated we had 80% power to detect the small to medium effect (Cohen’s *d* = 0.43; ɑ = 0.05) of cardiac cycle timing on cardiac and visual processing observed in a previous pilot study. Sample size hereby matches that of related foregone work^15^. Volunteers completed a battery of questionnaires assessing State/Trait Anxiety^28^, depressive symptoms^29^, body awareness^30^ and impulsivity^31^. All participants scored within the normal range.

### Ethics statement

Procedures were approved by the ethics committee of the Ludwig-Maximilian University Munich in accordance with the Declaration of Helsinki (BMF 1991; 302; 1194). All participants gave informed consent and were compensated for their participation (at the standard rate of 9€ per hour).

### Stop signal distractor task

Participants completed a stop signal paradigm in which their task was to respond quickly and accurately to the appearance of a go-cue. Each trial began with a right and left facing black arrow, presented in the centre of the screen. After a variable interval of 2.9–4.5 s, the go-cue appeared in the shape of either the right or left arrow lighting up green (directionality counterbalanced). The go-cue was shown for a fixed response time window of 1.5 s and followed by a feedback screen. Feedback was presented for 1 s and consisted of a green tick mark for correct (hits) and a red cross for incorrect (commission and omission errors) responses. Participants responded with a left [‘A’] or right [‘L’] button press on the keyboard respectively using the index finger of their left or right hand. The stop signal (the border of the screen lighting up blue) succeeded the go-cue in one third of all trials, for which participants were told to withhold their response. It was shown on screen for a maximum time window of 1 s or until the participant made a response. To maintain stop-signal performance at 50%, the stop signal delay (SSD) (i.e. the time that elapses between the appearance of the go-cue and the onset of the stop signal), was adjusted on a trial-by-trial basis by means of a staircase tracking algorithm. A correctly withheld response added 50 ms to the SSD while an incorrect response reduced the SSD by 50 ms. The baseline SSD for the first stop trial was set to 200 ms, the maximal SSD increase was set to 750 ms. Separate tracking algorithms were used for each of the three experimental conditions. In two of three conditions, participants were presented with distracting information in the time leading up to the appearance of the go-cue and subsequent stop-signal. These distractors were coupled to the cardiac cycle. As such, the time window of the arrow display was chosen to allow the recording of a minimum of three and a maximum of five heartbeats before the go-cue appeared. As heart rate is variable, the minimum length of a trial was 5.4 s (go-cue appears at 2.9 s at an average of 3 heartbeats) and the maximal length was 7s (go-cue appears at 4.5s at an average of 5 heartbeats). We conducted extensive control analyses to ensure trial length and distractor movements as a function of heart rate did not differ between go and stop trials or between experimental conditions (see “Results” in section).

The distracting information presented in conditions 1 and 2 consisted of 16 black dots (diameter 17 mm) which moved continuously across the screen with a velocity of 0.08°. In condition 1, the onset of a directional change in dot movement was set to coincide with the systole of the cardiac cycle (+ 290 ms post R-peak). In condition 2, the onset of a directional change of dot movement coincided with the cardiac diastole (+ 0 ms post R-peak). In condition 3, no distracting dots were shown. Dots that moved out of the confines of the screen immediately appeared on the opposite side. Their location shifted from [x, y] to [-x, -y] to ensure the number of dots on the screen remained constant. The dots’ movement direction changed dynamically, deviating randomly by plus 60–300° from their original motion. The absolute difference from foregone motion was set to a minimum of 60°. To ensure there were no dependencies between cardiac distractor timing and timing of subsequent stimuli each trial ended with an inter-trial interval of 500 ms.

**Fig. 1:**
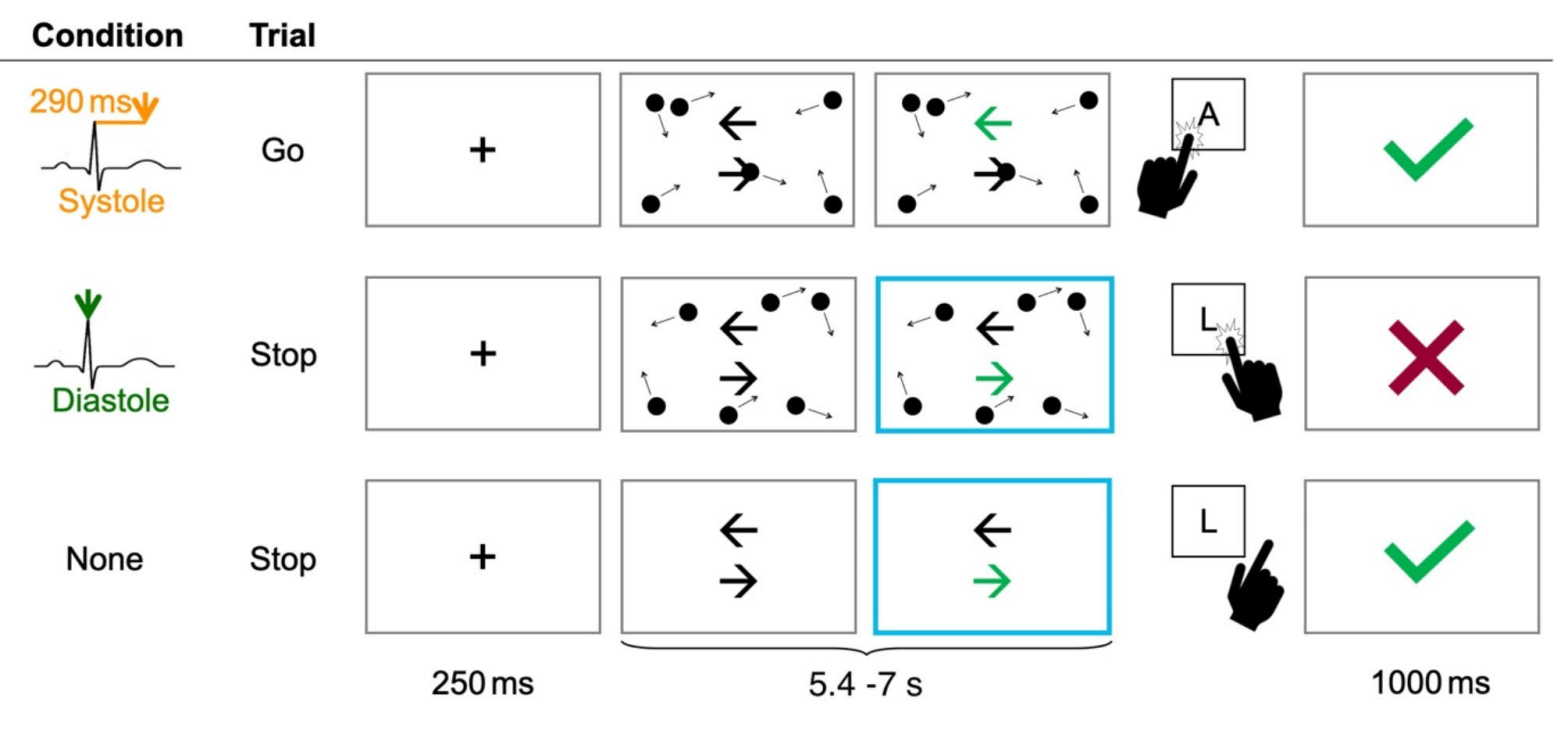
Overview of the timings and three experimental conditions (1: systole bound distractors; 2: diastole bound distractors; 3: no distractors) of the stop signal task. Depending on several factors (type of trial, length of the stop signal delay) the timing of the distractor window varied between 5.4 and 7 s. However, each time window included a minimum of 3 heartbeats/distractor movements.

The task comprised a total of 450 trials. These were presented as 3 blocks (one for each of the three distractor conditions) with 150 trials each (100 go, 50 stop). Our previous work indicates that cardiac effects on external perception take time to manifest. We therefore chose to present our conditions as blocks rather than trial by trial variations, similar to our forgone work^12,17,32^. To avoid order effects the blocks were counterbalanced across participants. Participants took a self-paced break in between each block and completed 9 practice trials before commencing the main experimental session. See Fig. 1 for an overview of the trial sequence.

### ECG recording and stimulus interfacing

**Fig. 2:**
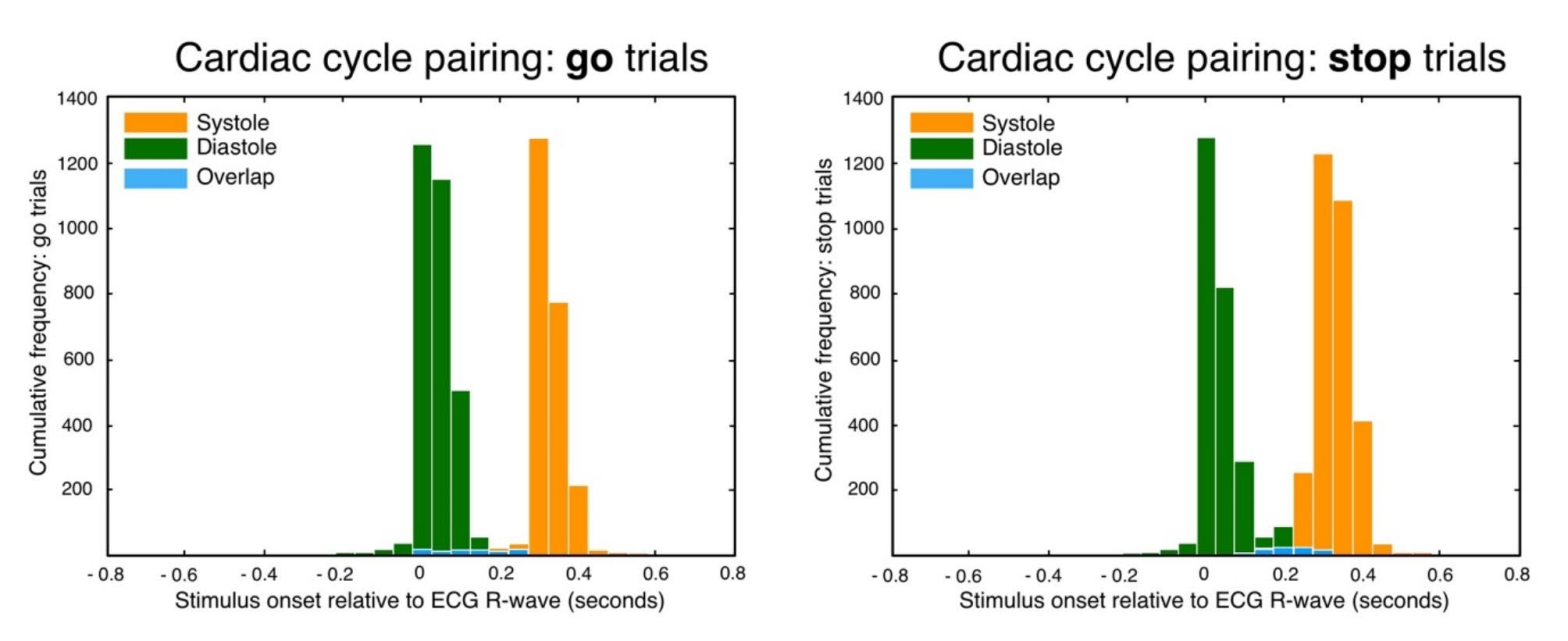
Distribution of event-trial timings relative to the R-wave peak in 50 ms time bins. Light blue indicates the overlap of diastole and systole trial timings which was minimal for both go and stop trials.

The ECG signal was recorded at the rate of 500 Hz from two bipolar electrodes placed below the left clavicle and right pectoral muscle. The ground electrode was positioned below the right clavicle. To interface distractor movement with different points in the cardiac cycle, we used RecView (Brain Vision GmbH)^33^ physiological recording software. RecView performs online R-peak detection and communicates this to the presentation software. To establish the precision of event timing within the cardiac cycle, we used in-house Matlab scripts to extract stimulus onsets from the EEG trace and calculate their relative timing to the R-wave peak determined from the offline ECG recording. The precision of event timing within the cardiac cycle was such that > 90% of trials were within 200 ms of the intended timing for both systole and diastole hereby mirroring the precision of foregone work^14^. For cardiac systole, distractor change of motion occurred at a mean timing of 296 ms (go-trials; SD = 68 ms) and 294 ms (stop-trials, SD = 71 ms) relative to the R-peak. For cardiac diastole, change of motion occurred at a mean timing of 3 ms (go trials, SD = 65 ms) and 4.5 ms (stop-trials, SD = 72 ms) relative to the R-peak. The distribution of cardiac cycle timings can be seen in Fig. 2. For subsequent HEP calculation, ECG data were offline band-pass filtered between 1 and 40 Hz (basic finite impulse response filter, Hamming windowed).

### EEG recording and processing

EEG was recorded at a sampling rate of 500 Hz from 64 active electrodes (BrainProducts, ActiCap) and one additional ground electrode (placed between FP1 and FP2), positioned according to the international 10–10 system. The FCz electrode was used as online reference. Using Fieldtrip, offline data were filtered (high-pass: 0.1 Hz, low-pass: 40 Hz) and re-referenced to the average of all electrodes. We performed independent component analysis (ICA) to identify and remove components unrelated to neural activity, such as eye movements and blinks. On average, we removed 2.4 ± 1.02 components per participant. Artefact free data was then obtained by back-projecting the remaining components onto the scalp electrodes.

We extracted electrophysiological activity related to cardiac and visual distractor processing, motor performance and feedback saliency. For the HEP, we segmented the data from − 100 to 1000 ms around the first three heartbeats recorded at the start of the trial sequence. This was done to avoid motor artefacts in the EEG trace as the go-cue and a subsequent button press did not occur until after a minimum of three heartbeats had been recorded. Accordingly, for visual evoked potential activity (VEP), we segmented the data from − 100 to 1000 ms around the first three movement triggers. For condition 1 (systole distractor movement), this meant cutting the data at 290 ms post R-peak. For condition 2 (diastole distractor movement) and condition 3 (no distractors), this meant segmenting the data as done for the HEPs (at 0 ms post R-peak). For motor performance on go-trials, we segmented the data from − 100 to 1000 ms around the onset of the go-cue. For motor inhibition performance in response to the stop-signal, we segmented the data from − 100 to 750 ms around the appearance of the stop-signal. For activity related to feedback processing, we cut the data from − 100 to 1000 ms around the four different feedback cues (correct go-trial: hit, incorrect go-trial: miss; correct stop-trial: rejection, incorrect stop-trial: commission). In all instances, we used the − 100 ms before appearance of the event trigger for baseline correction. After segmentation, we removed all segments in which activity exceeded ± 100 µV; this led to an average exclusion rate of 1.86% (SD = 3.72).

**Fig. 3:**
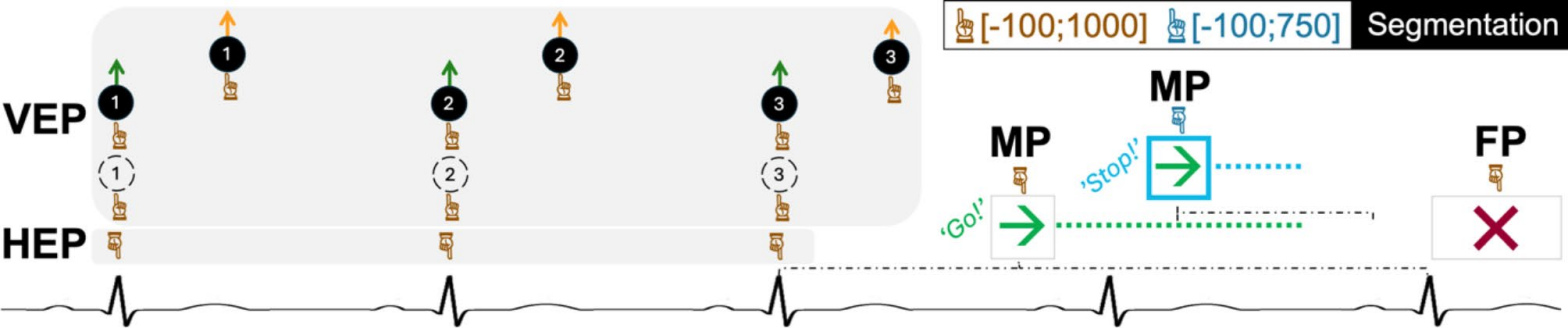
Data segmentation for analysis of event-related potentials. For each trial, data segments of interest were of length − 100 to 1000 ms around an event (marked by beige finger), except for motor processing of the stop signal (marked by blue finger) for which segments of -100 to 750 ms were extracted. To note, visual distractors were displayed throughout the whole trial up to feedback screen onset; however, visual evoked potentials of movement onsets (systole condition, yellow: 290 ms following R-peak; diastole condition, dark green: at R-peak; control condition without distractors, dashed black: at R-peak) were only analysed for those preceding go signal appearance (in total, three onsets). Similarly, heartbeat evoked potentials were only analysed for the first three heartbeats. The go signal (light green arror) appeared at random between the third and fifth heartbeat (lower.

dash-dotted black line); the stop signal (blue frame) appeared 200–750 ms after go signal onset (upper dash-dotted black line), dependent on stopping success in previous trials. In case no button was pressed, go cue display (dotted light green line) in go trials ended after 1000 ms, go and stop cue in stop trials disappeared once the stop cue had been displayed for 1000 ms (dotted light blue line).

VEP = visual-evoked potential, HEP = heartbeat-evoked potential, MP = motor-evoked potential, FP = feedback-evoked potential. unit = ms.

### Statistical analysis

For electrophysiological data, we determined ERP morphology and time windows of interest using a mass univariate permutation procedure following the principles described in Maris and Oostenveld^34^ and implemented in Fieldtrip. EEG data for the segmented time windows (0–1000 ms/ 0–750 ms) across all 64 electrodes was submitted to a repeated-measures, two-tailed permutation test. For each sample, i.e. a 2 ms time window resulting from the natural sampling rate of the data, we identified neural phenomena that differed for the main effect of systolic vs. diastolic distractor coupling and calculated point-estimate statistics (t-values using dependent samples t-tests) associated with this main effect. Samples were cluster-based on temporal and spatial adjacency (any electrodes within 5 cm of one another were considered spatial neighbours). Cluster-level statistics were calculated by taking the sum of t-values within each cluster and obtaining the test statistic largest in absolute value within each cluster. Significance probability was calculated via the Monte-Carlo method. For this, we created two new datasets by shuffling trials between both conditions and calculated the maximal value of cluster summed t-values resulting from their comparison. We permuted the dataset in this fashion 10,000 times. Across each permutation, the maximal test-statistic was logged, providing a distribution of maximal values obtained under the null hypothesis. We determined the p-value by calculating the proportion of random comparisons that produced a larger test statistic than the one originally observed. We selected all samples whose maximal t-values exceeded the critical alpha level of 0.05 for subsequent analysis.

Behavioural data consisted of accuracy scores (omission errors for go-trials, commission errors for stop-trials), median go-trial reaction times, median stop signal delay (SSD) and estimated stop signal reaction times (SSRT) to each of the three experimental conditions (systole distractors, diastole distractors, no distractors). SSRTs were estimated using the integration method^35^. Here, n go reaction times are rank-ordered and the SSD subtracted from the go reaction time corresponding to the n*probability of responding on stop trials. We used the specific SSD calculated by the staircase tracking algorithm for each condition to estimate the individual SSRT value for systole distractors, diastole distractors and no distractors respectively.

We analysed ERP amplitude (mean amplitude within the time window of interest determined by the permutation test), behavioural data as well as the link between ERP activity and behavioural performance using linear mixed effect models implemented in R^36^ (packages lme4 and lmerTest). For HEP and VEP analysis, the fixed effects consisted of HEARTBEAT/MOVEMENT NUMBER (1 vs. 2 vs. 3) and DISTRACTOR TYPE with either three (HEP: systole vs. diastole vs. none) or two levels (VEP: systolic vs. diastolic) to account for the absence of visual distracting input during condition 3. For neural activity to the go-cue, stop-signal and feedback as well as for behavioural values, the model included DISTRACTOR TYPE (systolic vs. diastolic vs. none) as a single fixed effect. To qualify the effects of ERP amplitude on inhibitory behavioural performance, the model fitted to the data used behavioural indices (SSD, SSRT) as the dependent variable and DISTRACTOR TYPE (systole vs. diastole vs. none) as well as ERP amplitudes (continuous) as fixed effects. For all models, the intercept was fit to zero and ɑ was set to the conventional level of 0.05. For the models exploring the interaction between ERP activity and behavioural indices, the random error term allowed for random intercepts of participants with ERP amplitude and distractor type nested within participants. For models exploring heartbeat processing, motor potentials and feedback processing the no distractor condition was set as the reference level. For the model on visual evoked potentials, the systole distractor condition was set as the reference level. As an additional control, we also added the fixed effects of trial length (mean length for go and stop trials) as well as heart rate, and heart rate variability (as indexed by the root mean square of successive differences, RMSSD for the 0–2.9 s time window before onset of the go-cue for each of the three experimental conditions) to each of the models.

### Control measures

Our experimental design tailored the presentation of stimuli to participants’ individual heartbeats. We therefore conducted a series of control analyses to ensure its implementation resulted in equal parameters across conditions.

First, we extracted the mean trial length for go and stop trials as well as heart rate and heart rate variability (as indexed by the root mean square of successive differences, RMSSD for the 0–2.9 s time window before onset of the go-cue for each of the three experimental conditions), and added those as control parameters to our Multiple Linear Models investigating Behavioural Performance as well as HEP, VEP, motor and feedback evoked cortical activity.

Further, we post-hoc coded go signals and stop signals according to their onset within the cardiac cycle to investigate whether cardiac phase (systole: R-peak to end of T-wave; diastole: end of T-wave to end of RR-interval) of those commands affected motor and feedback ERP activity (go signals: systole = 211, diastole = 239; stop signals: systole = 229, diastole = 221; n per condition). We added cardiac phase of go and of stop signals each as fixed factor to the models on motor and feedback processing.

To test whether cognitive rather than physiological cardiac effects governed our HEP and VEP results we extracted peak R-wave and mean T-wave expression (i.e. area under the curve) during the ~ 4600 ms time window in which participants viewed the arrow display. After filtering and R-peak detection, data was loaded into Brain Vision Analyser and epoched from − 150 to 150 ms around the R-peak and from 240 to 340 ms after the R-peak for R-peak and T-wave activity respectively. The Peak Information Export function was then used to extract the R-peak value within a ∓ 1 point interval around the peak. T-wave activity was exported using the area export function. We then compared peak R-waves and mean T-wave activity across the three different experimental conditions. We found no differences in their expression between the systolic linked distractor, the diastole linked distractor and the no distractor condition which indicates that HEP and VEP amplitude across these conditions manifested independently of potential differences in cardiac parameters.

In this experiment, we linked distractor movement to distinct points of the cardiac cycle. This had two consequences for our VEP and HEP data which we addressed in our control analyses. Firstly, for the diastole and no distractor condition VEP and HEP activity were time-locked to the same event. To address this issue and ensure that visual and heartbeat components did not infringe upon one another, we used independent component analysis to identify and remove their respective activity from the other’s data trace. Due to our experimental manipulation, both VEP and HEP activity had a high alignment with the time course of the ECG. We therefore computed the coherence between the ECG and the expression and time course of each component. We then generated an output of the topography, time course and average expression of the first 30 components showing the highest symmetry to the ECG signal. To delete the VEP, we searched these components for occipital as well as fronto-central topographies reflecting the detection of movement onset and for temporal distributions related to the perception of coherent motion^37–39^. We identified an average of 2.1 components and deleted these from the data trace used for HEP analysis. To delete the HEP, we searched for topographies across the vertex whose shape matched the flattened curve of the generally reported HEP^40,41^. This led to an average identification of 1.2 components which we removed from the data set used for VEP analyses. To test whether the exclusion of those relative components was successful, we extracted the grand average waveform across all participants and electrodes from each dataset and checked that each grand average wave in the time window from 0 to 1000 ms around the respective movement trigger showed no residual VEP or HEP activity. To ensure our conditions were treated equally, we performed this deletion for all three experimental conditions in their respective time windows. We ran our primary ANOVA across both control datasets and compared the results to our primary analysis to ensure the presence of additional ERP activity did not impact our VEP and HEP results (see supplementary material for control analyses and for corrected vs. uncorrected waveforms). Because there was a clear demarcation between VEP and HEP activity (200 to 300 ms vs. 600 to 700 ms respectively) we report uncorrected waveforms in our primary analysis.

Secondly, as the data segments for the VEP were cut around the movement triggers, the data segment for the systole VEP comes from a later time window (+ 290 ms post R-peak) than the VEP segments for the diastole and no distractor conditions (cut at 0 ms post R-peak). Those segments may therefore be differentially affected by the cardiac field artefact (CFA), an electrical field produced by the contraction of the heart-muscle as well as by effects of blood circulation. We therefore followed an established procedure^42^ to estimate the cardiac artifact in the EEG separately for systole and diastole trials. For this, we placed random triggers over cardiac cycles during the period of distractor encounter. Subsequently, we classified the arbitrary triggers as systole or diastole depending on the position of the trigger in the cardiac cycle. After this classification, data were segmented around these triggers (-1000 to 2000) and averaged separately for systole and diastole to estimate the cardiac field artefact separately for both kinds of trials for each EEG channel per subject. These signals were baseline-corrected 100 ms before the onset of the arbitrary triggers. To prevent any possible cardiac and pulsatility effects on the data, we subtracted the mean systole and diastolic artefacts from the data trace used for VEP analysis for all three conditions (see supplementary materials for uncorrected waveforms and additional control analyses). Because we report VEP activity in a time window very close to the CFA and because the CFA is unequally present across the experimental conditions, we in this instance, report datasets corrected for the CFA in our primary analysis.

## Results

### Behavioural performance

The model for median go-trial reaction times found no significant effects of distractor type (all p_s_ > 0.21). Investigation of median reaction times showed that participants were faster at responding when no distractors were present (453.9 ms) compared to the presence of diastole (465.3 ms) and systole (462 ms) linked distractors. However, this did not reach significance within the model. In contrast, for median SSDs Model 1 including the main effect of distractor type showed a parsimonious approximation of the data (Akaike information criteria [AIC] = 785.34, log likelihood value [LLV] = -372.13). An analysis of deviance calculation for this model revealed a main effect of distractor type (^2^ = 20.03, *p* < .001) showing that SSDs increased by 0.274 units from a no distractor to a diastole distractor trial (*p* = .007) while no such increase took place from the transition of a no distractor to a systole distractor trial (*p* > .93). Similarly, for SSRTs we observed a parsimonious model for the main effect of distractor type (Akaike information criteria [AIC] = 791.33, log likelihood value [LLV] = − 339.07) which showed that SSRTs significantly increased by 0.301 estimated units from a no-distractor to a diastole distractor trial (*p* = .034). Again, no such difference emerged for the transition from no-distractor to systole trials (*p* > .72). Behavioural results hereby demonstrate that inhibitory processes in the stop-signal task were significantly impaired by the presence of diastole coupled distractors which adversely altered behavioural performance from no distractor trials. Conversely, systole coupled distractors did not impact inhibitory functioning to the same degree. An overview of behavioural scores and physiological measures can be viewed in Table 1. The main effects of trial length, go- and stop-cue window, heart rate and heart rate variability did not reach significance in either the SSD or the SSRT model, neither did any of the interaction terms between distractor type and these control variables. Data hereby indicate that cardiac parameters and trial length did not affect behavioural outcomes.

**Table 1:** Overview of behavioural scores on the Stop Signal Task as well as physiological cardiac measures.

	Systole coupled	Diastole coupled	No-distractors
Go hits	93.1 (2.3)	89.7 (1.5)	91.6 (2.7)
Go omissions	6.9 (0.3)	10.3 (0.7)	8.4 (0.6)
Stop commissions	23.7 (1.1)	26.8 (1.8)	25.2 (2.3)
Go RTs (ms)	462 (15.1)	465.3 (11.8)	453.9 (9.08)
SSRT (ms)	**216.2 (36.2)**	**231.9 (33.3)**	**211.4 (26.8)**
SSD (ms)	**340.8 (11.4)**	**322.9 (10.4)**	**342.3 (12.6)**
MeanHB (bpm)	58.2 (11.2)	58.6 (12.6)	58.3 (9.3)
MeanHRV (ms)	53.5 (8.9)	52.9 (10.7)	55.7 (13.4)

We observed significantly shorter stop signal delays (SSDs) for diastole bound distractor trials relative to systole and no distractor trials. We further observed significantly longer stop signal reaction times (SSRTs) for diastole relative to systole and no distractor trials (significant measures in bold).

Significant values are in [bold]

We further correlated behavioural indices (SSD, SSRT and reaction times for the three conditions) with participants’ scores for depressive symptoms, state/trait anxiety, body awareness and separate subscales for impulsivity (negative urgency, premeditation, perseverance, sensation seeking). We found no significant correlations between behavioural performance and scores indexing anxiety and depression (see supplementary Table 1). We also did not find a significant relationship between behavioural measures of inhibitory control and self-report values of impulsivity (see see supplementary Table 2).

### Heartbeat processing during distractor movement

**Fig. 4:**
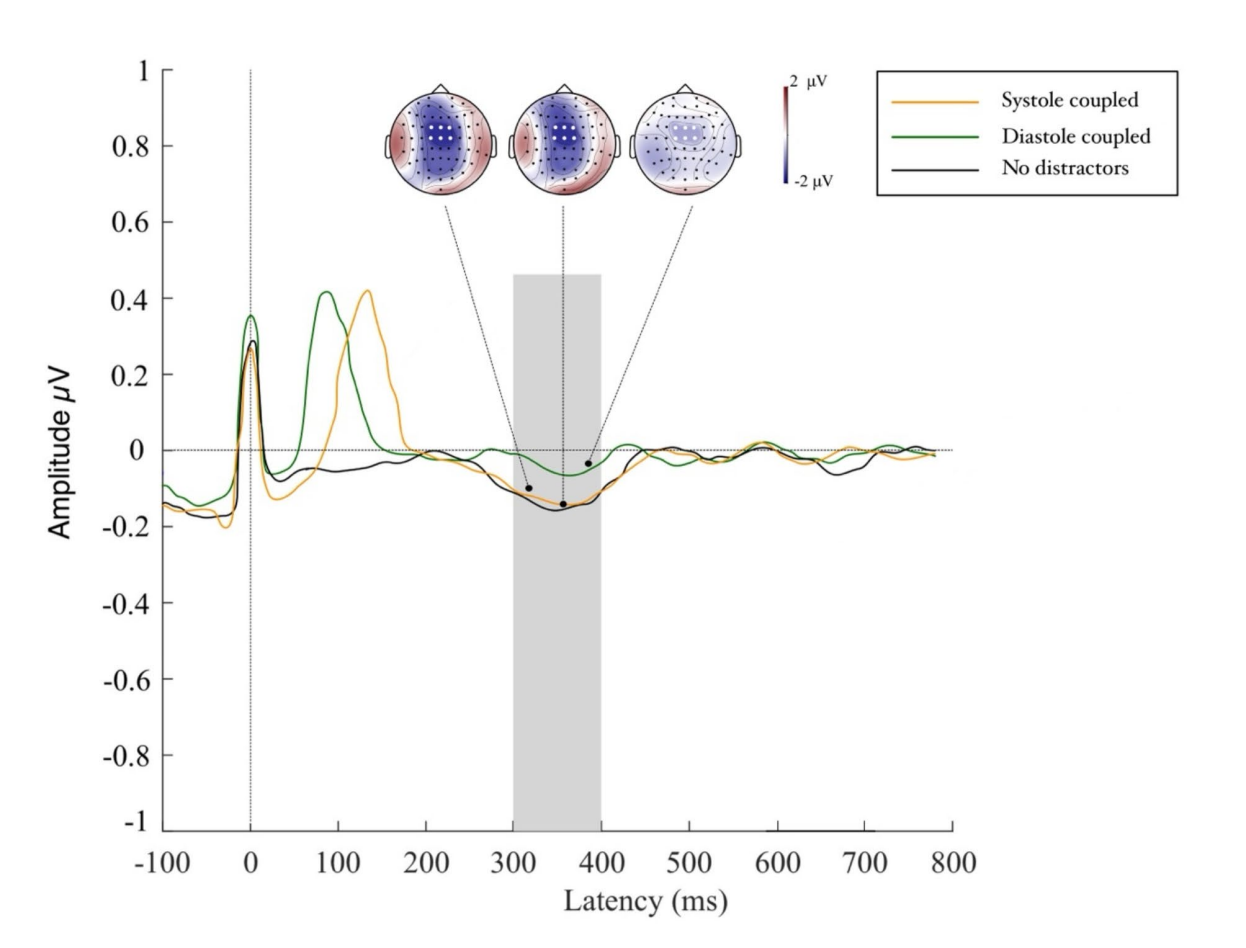
Amplitudes and scatter graphs of the Heartbeat Evoked Potential manifesting at 300–400 ms post R-peak. Differences in HEP activation hereby occur after perception of the moving triggers (at R-peak + 0 for diastole and R-peak + 290 ms for systole trials).

The permutation test contrasting systole with diastole distractor conditions returned a significant effect across the fronto-central region (FC1, FCz, FC2, C1, Cz, C2) which manifested from 300 to 400 ms following the R-peak marker (see Fig. 3). Across this electrode cluster, the Model 1 including the main effects of distractor type and heartbeat number showed a more parsimonious fit of the data (Akaike information criteria [AIC] = 803.07, log likelihood value [LLV] = -411.72) compared to Model 2 (including main effects and interaction term). An analysis of deviance calculation of this model revealed a main effect of distractor type (^2^ = 23.93, *p* < .001) highlighting that HEP amplitude decreased by 0.335 estimated units from a no-distractor to a diastole distractor trial (*p* < .001; see Fig. 4) while no such difference appeared for the transition from no distractor to systole trials (*p* > .43). The main effect of heartbeat number did not reach significance (*p* > .58). Similarly, the main effects of trial length, heart rate and heart rate variability did not reach significance in the model and neither did any of the interaction terms including these variables (all p_s_ > 0.15). Results therefore indicate that cortical cardiac processing was altered in the diastole coupled distractor condition compared to when distractors were either coupled to cardiac systole or not present at all.

### Visual processing of distractor movement

**Fig. 5:**
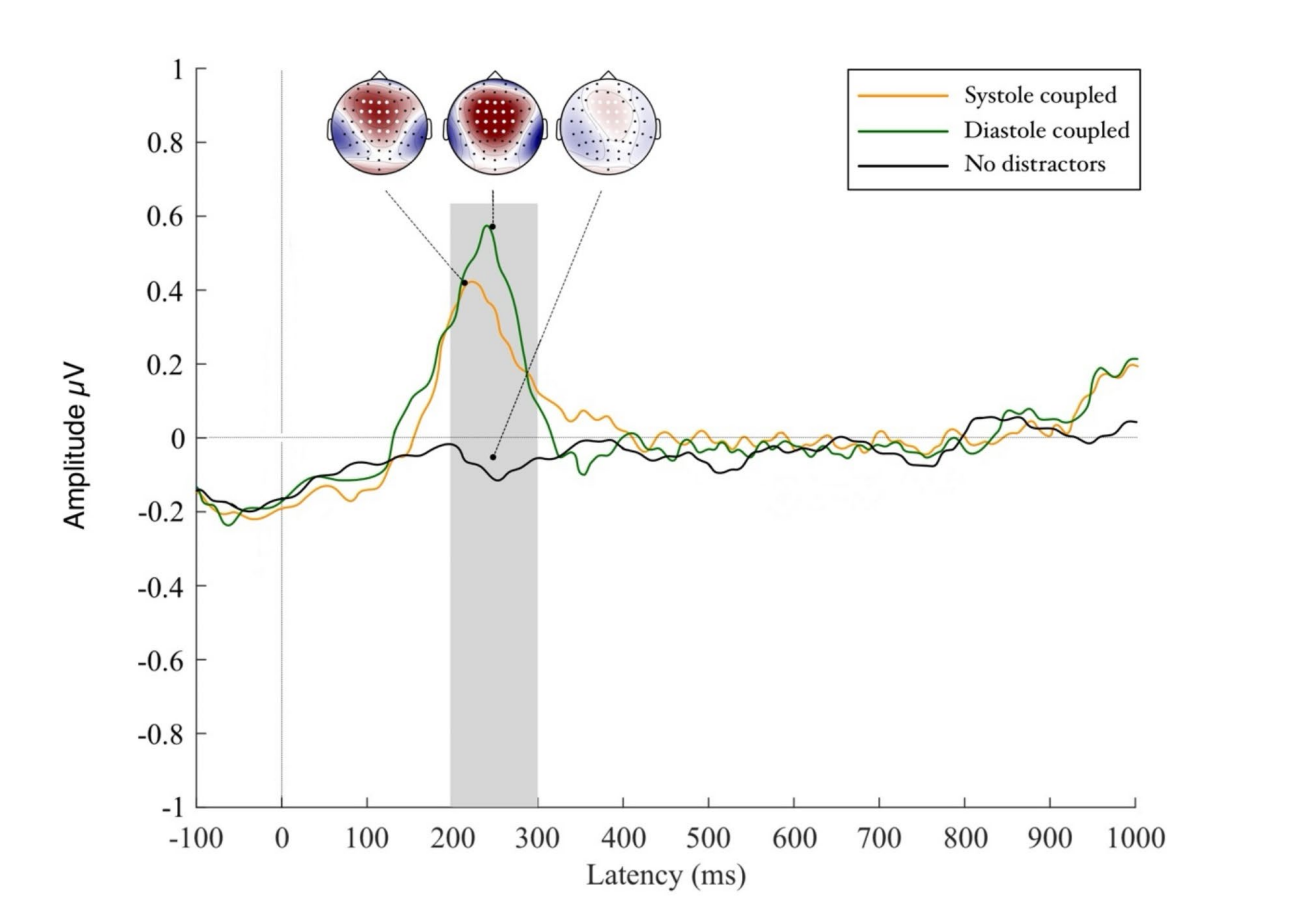
Amplitudes of the P2 potential in response to viewing the moving distractors. P2 amplitudes for systole distractors were lower than those for diastole distractors, highlighting efficient suppression of distracting information in systole trials. To illustrate that no visual distraction was present in our control trials we included the no distractor condition in this graphic.

Results of the permutation test revealed a large area of interest across fronto-central regions (F3, F1, Fz, F2, FC3, FC1, FCz, FC2, FC4, C3, C1, Cz, C2, C4, CPz, CP1, CP2) which reached significance at 200–300 ms following movement onset, thus suggesting a P2 event-related potential component (see Fig. 4). Across this electrode cluster we compared models incorporating the single main effects as well as the interaction term between movement number by distractor type. Model 1 including purely the main effects provided the most parsimonious approximation of the data (Akaike information criteria [AIC] = 698.97, log likelihood value [LLV] = -393.71). An analysis of deviance of this model revealed a main effect of distractor type (^2^ = 18.02, *p* < .001) highlighting that VEP amplitudes increased by an estimated 0.320 units from a systole to a diastole distractor trial (see Fig. 5). The main effect of movement type did not reach significance, neither did the control variables of trial length, heart rate and heart rate variability (all p_s_ > 0.38). Results hereby indicate suppressed cortical activity towards visual distractor input when it co-occurs with strong cortical heartbeat feedback at systole which significantly differs from the elevated cortical response evoked by diastole-linked distractors. We are further able to show that VEP activity was not affected by cardiac parameters and trial length.

### Motor potential to the stop-signal

**Fig. 6:**
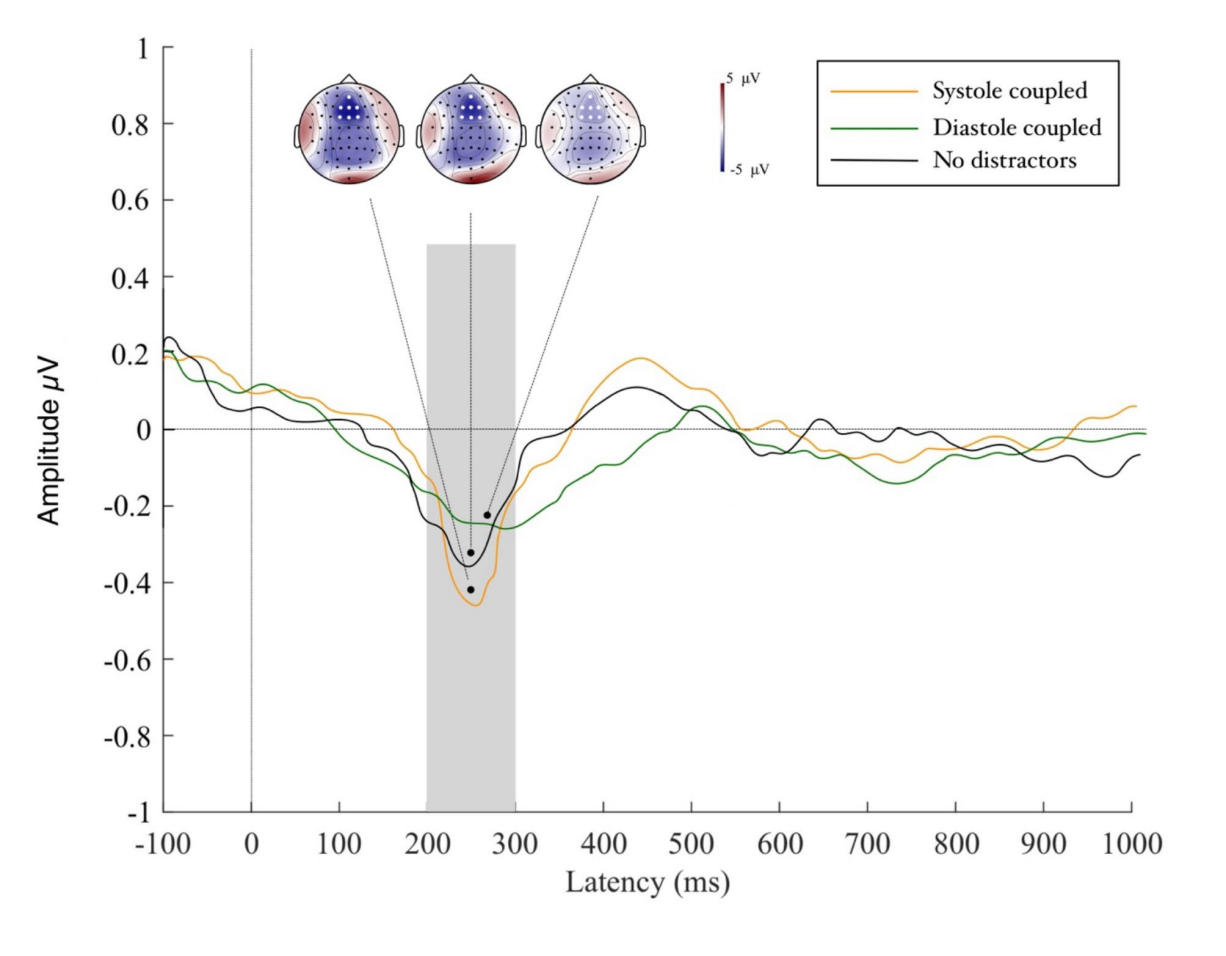
Amplitudes of the N2 potential manifesting in response to stop-signal onset. Amplitudes of the N2 are higher for systole relative to diastole trials, suggesting greater allocation of cognitive control processes.

For motor activity in response to the go-cue in go trials, our permutation test returned no significant clusters for the comparison of diastole vs. systole linked distractors. For motor activity to the stop-signal, the model revealed a significant area of interest across the fronto-central region (AFz, F1, Fz, F2, FC1, FCz, FC2) which manifested 200 to 300 ms after onset of the stop-signal, suggesting an N2 event-related potential (see Fig. 5). The model including the main effect of distractor type provided a good approximation of the data (Akaike information criteria [AIC] = 893.43, log likelihood value [LLV] = -386.04). An analysis of deviance calculation revealed a significant main effect of distractor type (^2^ = 8.91, *p* = .008) showing that N2 activity decreased by an estimated 0.203 units from a no distractor to a diastole distractor trial (see Fig. 6). No significant difference occurred for the transition from a no distractor to a systole distractor trial. Furthermore, neither the main effects nor the interaction terms of trial length, heart rate, heartrate variability or go-and stop-cue cardiac window reached significance. Our findings hereby show that motor performance on trials where distractors moved in time to the cardiac systole approximated that of trials in which no distractors were present. Conversely, we observed reduced cortical motor activity to the stop-cue when it succeeded diastole-coupled distractors. This effect was not impacted by trial or cardiac parameters.

### Feedback processing

**Fig. 7:**
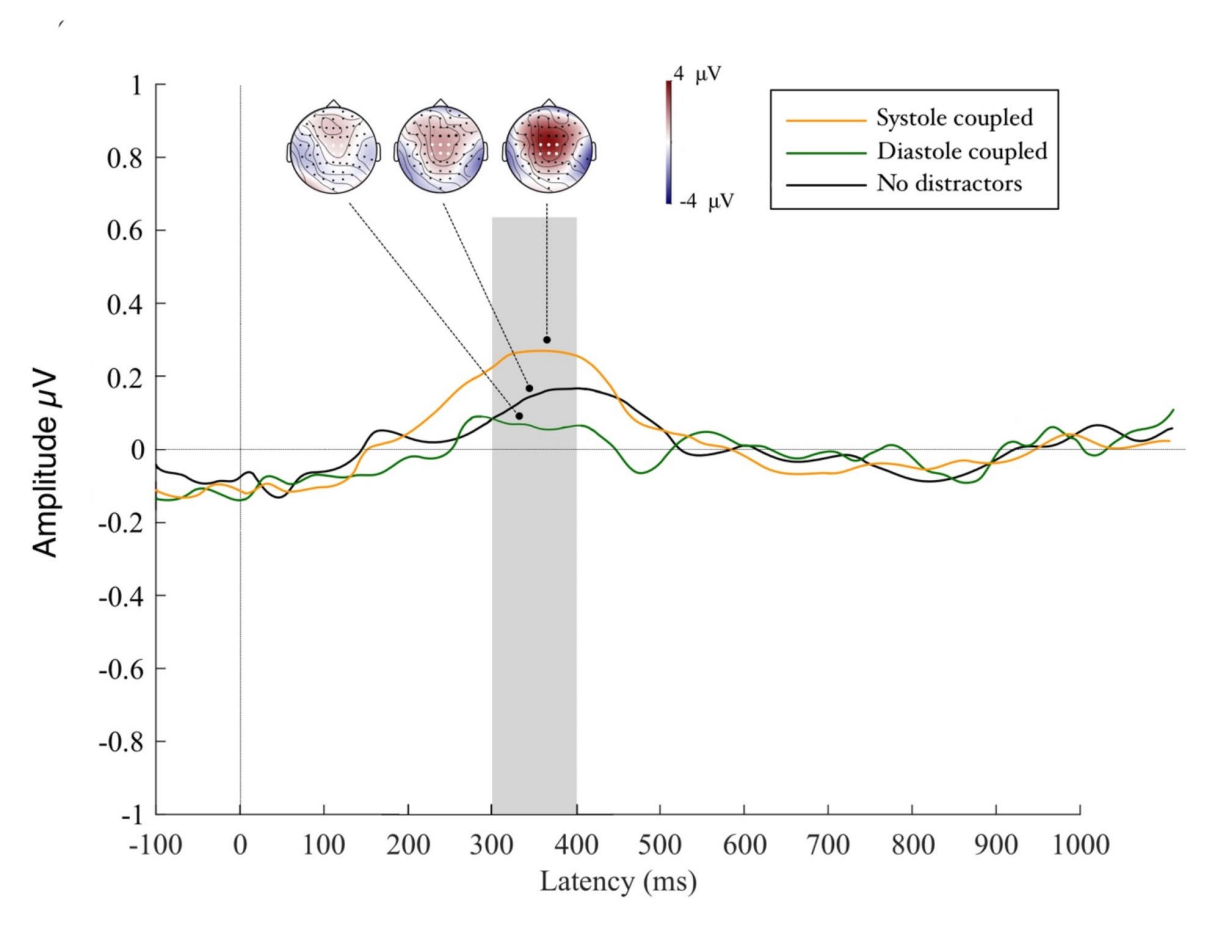
Amplitudes of the P3 potential in response to feedback for commission errors in stop trials. We observed higher amplitudes for systole trials relative to both diastole and control trials, suggesting negative feedback cues were processed with greater saliency when followed by performance under systole bound distraction.

Our permutation test returned no significant clusters for hits, misses or correct rejection feedback. However, for commission errors on stop-trials (i.e. feedback for an incorrectly provided response) we found a significant effect across a central cortical region (C1, Cz, C2, CP1, CPz, CP2) which manifested around 300 to 400 ms after onset of the feedback cue, thereby suggesting a P3 event-related potential component (see Fig. 7). Our model including the main effect of distractor type returned parsimonious fit of the data (Akaike information criteria [AIC] = 755.03, log likelihood value [LLV] = -364.84). An analysis of deviance revealed a significant main effect of distractor type (^2^ = 9.03, *p* = .006) highlighting that P3 activity increased by 0.438 estimated units from a no distractor to a systole distractor trial. No such difference emerged for the transition from a no distractor to a diastole distractor trial (*p* = .72; see Fig. 7). Similarly, the main effects of trial length, heart rate and heart rate variability did not reach significance in the model (all p_s_ > 0.42). Results hereby indicate increased cortical activity for processing the error signal on stop-trials following the presentation of systole-linked distractors which interestingly is higher even then trials in which performance was not impacted by the presence of distractors. Once again, the control variables of trial length, heart rate, heart rate variability and go-/stop-cue cardiac window did not reach significance in the model highlighting that feedback evoked activity was not impacted by these parameters.

### The relationship between ERP expression and behavioural performance

**Fig. 8:**
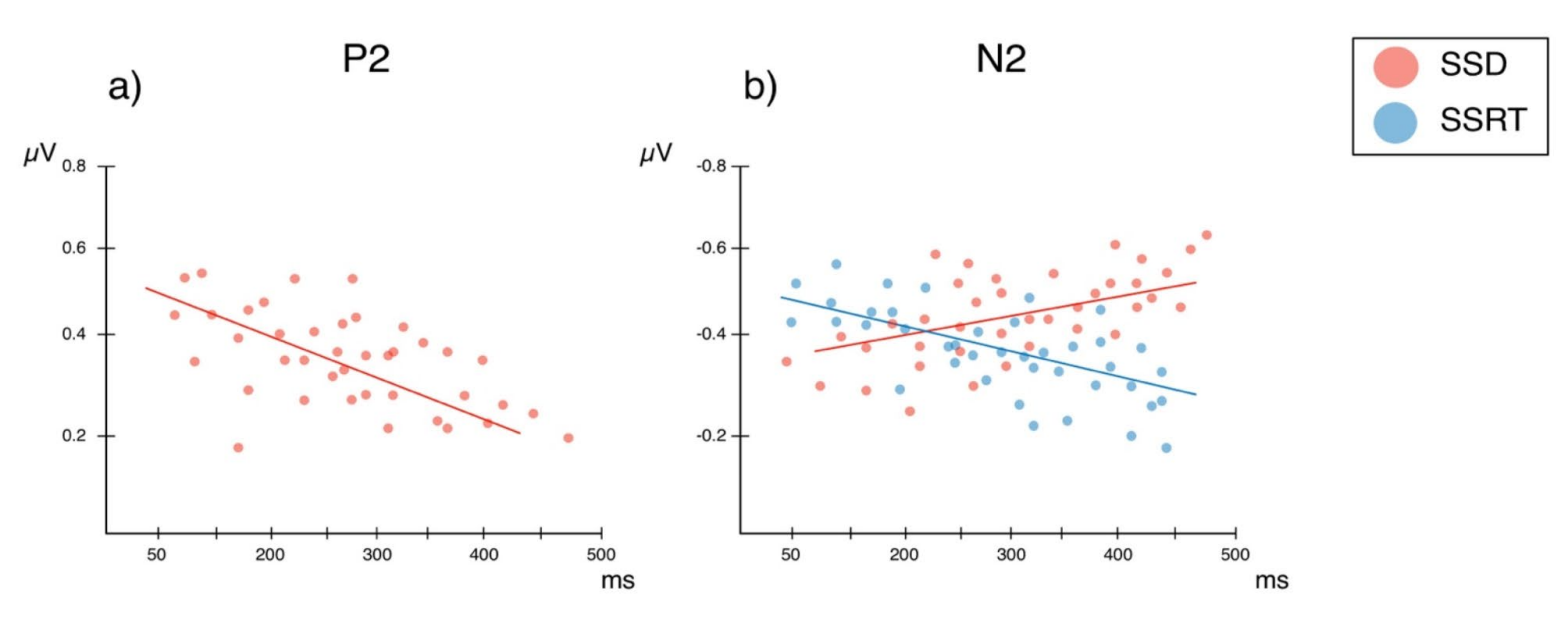
Relationship between behavioural scores (SSDs, SSRTs) for the diastole distractor condition. (**a**) Results found that higher P2 amplitudes led to significantly shorter stop signal delays (i.e. more impaired inhibitory performance). (**b**) Results showed that reduced N2 amplitude led to a significant increase in stop-signal reaction times (i.e. longer response times to the motor-command issued by the stop-signal) as well as a decrease in SSDs.

For SSDs, the models exploring HEP and feedback P3 amplitude alongside the fixed factors of distractor type returned no significant main effects or interactions (all p_s_ > 0.17). For the model including the P2 component evoked from processing the visual movement of distractors we compared the parsimony of Model 1 (MEs of P2 and distractor type; Akaike information criteria [AIC] = 865.34, log likelihood value [LLV] = − 347.23) and Model 2 (ME of distractor type alongside the two-way interaction of distractor type by P2 amplitude [AIC = 798.67, LLV = − 336.78]. Based on a comparison of the AIC and the LLV, we chose Model 2 as providing the most parsimonious fit for the data (^2^ = 23.46, *p* < .001). An analysis of deviance calculation for this model revealed a main effect of distractor type (^2^ = 20.03, *p* < .001). SSDs decreased by − 0.211 estimated units from a systole to a diastole distractor trial (*p* = .032). In addition, results revealed a P2 x distractor type interaction (^2^ = 9.11, *p* = .003). A simple slopes analysis of this showed that that stop-signal delays decreased significantly as a function of P2 amplitude in diastole distractor trials (− 0.352 estimated units; t_39_ = 2.75, *p* = .038; see Fig. 8a), highlighting that higher P2 activity in distractor trials coincided with an increased tendency to commit an erroneous motor response on stop-trials. For the model containing N2 activity towards the stop-signal, the second model including both main effects and the distractor type by N2 amplitude interaction provided the most parsimonious fit to the data [AIC = 778.03, LLV = − 322.73; ^2^ = 17.36, *p* < .001]. An analysis of deviance calculation revealed a significant distractor type by N2 amplitude interaction (^2^ = 7.63, *p* = .027). A simple slopes decomposition of this interaction found that for diastole distractor trials reduced N2 amplitude led to a significant decrease in SSDs (-0.402 estimated units; t_39_ = 3.07, *p* = .028; see Fig. 8b). Results thus show a significant link between reduced N2 amplitude and an increased tendency to provide a motor response in diastole distractor stop trials.

For SSRTs the models exploring HEP, visual P2 and feedback P3 amplitude alongside the fixed factors of distractor type returned no significant main effects or interactions (all p_s_ > 0.23). For the model containing N2 activity evoked by the stop-signal, the second model including both main effects and the distractor type by N2 amplitude interaction provided the most parsimonious fit to the data [AIC = 748.03, LLV = − 382.43; ^2^ = 11.92, *p* < .001]. An analysis of deviance calculation revealed a significant distractor type by N2 amplitude interaction (^2^ = 8.67, *p* = .009). A simple slopes decomposition of this interaction found that for diastole distractor trials reduced N2 amplitude led to a significant increase in SSRTs (− 0.503 estimated units; t_39_ = 4.19, *p* = .012; see Fig. 8b). Results thus show a significant link between reduced N2 amplitude and a decreased ability to process the motor command of the stop-signal.

## Discussion

## Behavioural findings

In this study, we linked distracting visual information to different points of the cardiac cycle and studied its impact on inhibitory motor performance in a stop-signal task. We found that participants were significantly better at inhibiting their motor response when distracting, task-irrelevant information, which led attention away from relevant go and stop-cues, co-occurred with cortical heartbeat feedback at systole. While accuracy scores and reaction times for go-cues remained unaffected by our experimental manipulation, stop-signal delays were significantly longer for systole relative to diastole trials while stop-signal reaction times were significantly shorter. Moreover, stop-signal delays and stop-signal reaction times on systole trials were nearly equal to those on control trials in which no distracting information was presented. This underscores that participants were highly efficient at cancelling out interfering visual signals when those were bound to the systole of the cardiac cycle. In contrast, visual distractors coupled to the cardiac diastole could not be filtered out as effectively.

## Electrophysiological findings

### Visual distractor suppression is enhanced in systole trials

Our electrophysiological evidence sheds further light on the cognitive mechanisms underpinning this behavioural result. We observed a significantly reduced P2 component for processing distractors at systole relative to diastole. Furthermore, we found a significant association between enhanced P2 amplitude on diastole distractor trials and shorter stop-signal delays which highlights that electrophysiological processing in response to diastole bound distractors is linked to the impaired inhibitory performance we observed on these trials. P2 components manifesting over fronto-central regions have been linked to the processing of movement onset^37,38^. Our results thus highlight that less cognitive processing of distractor movement occurred at systole which, in line with our behavioural findings, further suggests that distractors could be disregarded more easily when they occurred jointly with cardiac feedback to the brain.

### Motor control is reduced in diastole trials

Regarding neural markers of motor control, we observed that amplitudes of the N2 component coupled to the stop-signal were significantly reduced when visual distractors moved during cardiac diastole, compared to when they moved during cardiac systole or when they were absent. Here, we likewise observed a significant relationship between reduced N2 amplitude towards the stop signal in diastole trials and both measures of behavioural inhibition. Reduced N2 amplitude coincided with shorter SSDs indexing faster, more impulsive motor responses and with longer SSRTs highlighting lengthier reaction periods towards the appearance of the stop signal. Amplitudes of the N2 have been shown to reflect an awareness of disruptions to a motor sequence^43^ as well as successful anticipation of the stop-signal and have thus been interpreted as enhanced cognitive motor control that aides maintaining behavioural performance in light of this disturbance^44,45^. Our reduction of N2 amplitude in diastole trials may thus reflect reduced anticipation of the stop-signal. In contrast, when distractors moved during cardiac systole, we provide evidence that anticipation of the stop-signal and the necessary increase of motor control for correctly withholding the motor response in this instance is more effective, approximating the neural pattern of undisturbed performance.

### Feedback processing is enhanced in systole trials

Finally, we found that P3 activity in response to feedback for stop-trial commission errors was significantly higher for systole, relative to diastole and no distractor trials. Amplitudes of the feedback P3 have been linked to the attentional categorisation of salient outcome information^46,47^ as well as context updating, manifesting in situations in which one’s model of the environment must be updated^48^. Our previous work suggested that interoceptive processing can affect the efficacy with which feedback cues can be processed^17^. In line with these findings, our current results indicate that feedback for motor mistakes in stop-trials is processed with greater salience in trials where distracting information is coupled to cardiac systole compared to diastole. Interestingly, this is also the case for feedback processing following motor inhibition performance without any visual distraction, which may hint to the beneficial effects of presenting visual input aligned to cortical heartbeat feedback for feedback processing of commission errors. Combined, our results highlight a complex effect of cardiac phase on the interplay of visual selection efficiency and motor inhibition. Presenting distractors conjointly with cardiac feedback at systole allows for more efficient inhibition of irrelevant visual information during motor task performance. In turn, this seems to facilitate greater inhibitory motor control and anticipation of the stop-signal, as well as greater salience processing of error feedback. Results thus suggest that these cognitive mechanisms may give rise to the elevated behavioural performance we observe on systole relative to diastole distractor trials.

## Distractor suppression relative to cardiac predictive processing

Our findings correspond to past reports of facilitated visual selection efficiency at systole^49,50^. According to our findings, the brain seems more successful at suppressing distracting information when the latter is linked to the cardiac systole. Explanations for this phenomenon are rooted in the framework of predictive processing^51,52^, which suggests that cortical heartbeat processing occurs via a predictive process. The brain is thought to continuously construct and update an internal model of the body’s physiological state through an iterative process of Bayesian hypothesis testing whose goal it is to minimize the mismatch between this internal model and the afferent sensory input. The brain is thus assumed to anticipate the periodic signal of the heartbeat by generating priors of the heartbeat signal. These priors are then matched to afferent cardiac information in an effort to minimise extraneous sensory noise which could otherwise have adverse effects on the effective processing of external sensory information. Prediction errors that arise from a mismatch between priors and afferent input are utilised to either revise prior beliefs thereby inducing perceptual modifications or by performing a reflexive action that aligns priors with reality. The heartbeat specifically, is known to generate a lot of extraneous noise. It can affect tactile afferent output^22^, muscle spindle discharge^23^ as well as eye movement and interocular pressure^24^. The cardiac signal thus has the potential to significantly distort both the intake of information as well as fine-motor interactions with the environment. The brain is thus strongly incentivised to anticipate and cancel out the bodily side-effects produced by the heart muscle’s contraction. And indeed, earlier work has suggested that external stimuli bound to baroreceptor feedback at cardiac systole are automatically cancelled out as a side-effect of the predictive mechanism that effects the suppression of the sensory consequences of the heartbeat from awareness^9^.

## HEP results in light of a generalized suppression of cardiac (-bound) signals

Our HEP findings correspond to this idea of predictive cardiac suppression. Within the framework of interoceptive predictive processing, the HEP can be understood as a precision-weighting process of prediction errors associated with heartbeat sensations^20^. Studies to this effect have demonstrated that interoceptive attention to heartbeats results in increased HEP amplitude, a phenomenon ascribed to the amplification of prediction error weighting^53,54^. In our study, we observed reduced HEP amplitudes for distractor bound diastole trials, relative to HEP expression for systole bound distractors and control trials in which no visual distraction occurred. The reduced HEP expression at diastole may thus indicate an impaired cardiac precision-weighting mechanism of prediction errors which occurs on trials where there is a mismatch between a periodic external signal and cortical cardiac feedback. This may impact the successful suppression of cardiac side effects and in turn lead to the reduced inhibition of diastole bound distractors observed in the current study. Further findings which correspond to the idea of a generalised cortical suppressive mechanism at cardiac systole are provided by the work of Ren and colleagues^16^, who likewise explored the effect of cardiac phase on motor performance in a stop-signal paradigm. Contrary to the current design in which irrelevant information (distractors) were bound to the cardiac phase, Ren and colleagues paired the stop-signal with the cardiac cycle, thereby linking target relevant information to the heartbeat signal. Results showed impaired inhibitory performance for trials in which the stop-signal was linked to the cardiac systole. Electrophysiological analyses revealed a reduced P3 amplitude alongside elevated HEP activity. Similarly, Makowski and colleagues^55^ found participants were less likely to inhibit a systole linked motor action on a Go/NoGo task which simultaneously assessed reaction times, choice reaction times, inhibition and conflict resolution. These findings speak to a general suppression of systole linked information, which, on the one hand, facilitates performance when systole linked information is task irrelevant and, on the other hand, impairs performance when systole linked information is task relevant. Our current findings correspond well to this parsimonious account of the effects of cardiac phase on external signal processing.

## Cardiac suppression and dynamic allocation of cognitive resources

However, we must note that other studies have highlighted a more dynamic process. For example, Pramme and colleagues^10,11^ demonstrated that participants were better at selectively filtering out relevant from irrelevant (distracting) information when both were presented simultaneously at cardiac systole. Similar effects of elevated performance at cardiac systole have been reported for visuomotor processing where past work has shown that self-initiation of visual input by means of a motor action is preferentially timed to the cardiac systole^7^ and that visual motor cues appearing at systole are responded to with greater accuracy^14^ and perceived with greater saliency^8^. This rather suggests that the brain can utilize the window of cortical cardiac suppression to dynamically allocate cognitive resources to other processing domains. To note, our data likewise illustrates that suppression of visual distractor processing at systole, co-occurred with enhanced indices of motor control and, at a later stage, improved feedback processing of commission errors. Therefore, our data may also point to an effective re-allocation of processing resources during this window of cortical cardiac suppression.

## Cognitive resource allocation depends on task design and difficulty

In addition, several studies have illustrated that the cardiac phase effect on performance seems dependent on task difficulty and cognitive load. Larra, Finke, Wascher and Schächinger^56^ reported a sensorimotor conflict task in which participants exhibited faster reaction times at systole for congruent trials while incongruent systole trials produced slower reaction times. Similarly, Adelhofer and colleagues^57^ reported that systole coupling enhanced inhibitory performance on a face-word Stroop task for congruent trials whereas the effect was no longer present for incongruent trials. The potentially facilitating effects of cardiac phase may thus be highly dependent on the cognitive demands of the task and may differ based on task difficulty. Future work should thus investigate whether the observed phenomena arise from general cortical suppression at systole or whether the brain makes use of predictive cardiac suppression to selectively process task relevant information and whether this process breaks down for higher difficulty levels. For a stop-signal paradigm, this could be achieved by implementing a block design in which go-cues are linked to cardiac diastole and stop-signals to cardiac systole (and vice versa) to contrast effects of cardiac phase and relevance of aligned input to task demands. Difficulty levels could be increased by adding an increasing amount of distracting information similar to the current design.

## Conclusion

To conclude, our results add to our understanding of the effect of cardiac phase on external signal processing. We highlight the complex interplay between the way our brain integrates external and internal information by demonstrating enhanced visual selection efficiency for irrelevant visual information bound to cardiac systole during motor inhibition performance with subsequent downstream implications for motor and feedback processing. We hereby provide a significant contribution to a highly important field of research which is rapidly illuminating efficient predictive mechanisms that combine external and internal signal processing to holistically shape our embodied experience of the world.

### Probabilistic gender ratio of reference list

Recent evidence suggests that publications with female names at prominent positions in the author list are cited less often in the field of neuroscience than should be expected. To raise (self-)awareness and to work towards more equitable research practices, we here report our own citation practices. We extracted gender estimates (F = female; M = male) for the first and last authors’ names across all publications through both an automated service (https://gender-api.com) and a manual search for publicly available pronouns. Among the 50 cited publications and following the exclusion of self-citations (i.e. citing the first and/or last author of this work), 6% (*n* = 3) were FF, 10% (*n* = 5) were MF, 30% (*n* = 15) were FM, and 54% (*n* = 27) were MM.

To note, the applied procedure falls short of two aspects: (1) gender estimates and openly accessible pronouns may not be, in full, reflective of the respective individuals’ gender identities, and (2) gender is taken as dichotomous variable only, and thus, the procedure does not allow for monitoring gender representation in its full variety.

Dworkin, J. D., Linn, K. A., Teich, E. G., Zurn, P., Shinohara, R. T., & Bassett, D. S. (2020). The extent and drivers of gender imbalance in neuroscience reference lists. *Nature Neuroscience*, *23*(8), 918–926. https://doi.org/10.1038/s41593-020-0658-y.

## Electronic supplementary material

Below is the link to the electronic supplementary material.

Supplementary Material 1

## Data Availability

Data and materials of this study are archived online at: DOI 10.17605/OSF.IO/C6YT4.
