# Supplementary material for: The effect of cardiac phase on distractor suppression and motor inhibition in a stop-signal task
Source: Sci Rep. 2024 Dec 2;14:29847. doi: 10.1038/s41598-024-80742-2 (PMC11609284; doi:10.1038/s41598-024-80742-2)
Supplement: Supplementary file 1 — Supplementary Material 1 [file 41598_2024_80742_MOESM1_ESM.docx]

|  | RT-s | RT-d | RT-n | SSRT-s | SSRT-d | SSRT-n | SSD-s | SSD-d | SSD-n | BDI | STAI Trait | STAI State |
| --- | --- | --- | --- | --- | --- | --- | --- | --- | --- | --- | --- | --- |
| RT-s |  |  |  |  |  |  |  |  |  |  |  |  |
| RT-d | r = 0.314  **p = 0.049** |  |  |  |  |  |  |  |  |  |  |  |
| RT-n | r = 0.552  **p = 0.005** | r = - 0.572  **p = 0.039** |  |  |  |  |  |  |  |  |  |  |
| SSRT-s | r = - 0.424  **p = 0.006** | r = 0.340  p = 0.054 | r = - 0.342  p = 0.053 |  |  |  |  |  |  |  |  |  |
| SSRT-d | r = - 0.371  **p = 0.042** | r = 0.563  **p < .001** | r = 0.449  **p = 0.032** | r = - 0.320  **p = 0.048** |  |  |  |  |  |  |  |  |
| SSRT-n | r = 0.755  **p < .001** | r = - 0.439  **p = 0.013** | r = 0.375  p = 0.064 | r = - 0.468  **p = 0.030** | r = - 0.704  **p < .001** |  |  |  |  |  |  |  |
| SSD-s | r = - 0.467  **p = 0.002** | r = - 0.645  **p = 0.009** | r = 0.553  **p = 0.003** | r = - 0.374  **p = 0.036** | r = 0. 610  **p = 0.004** | r = -0.230  p = 0.053 |  |  |  |  |  |  |
| SSD-d | r = - 0.438  **p = 0.038** | r = -0.402  **p = 0.031** | r = - 0.621  **p = 0.003** | r = 0.514  **p = 0.018** | r = - 0.307  p = 0.058 | r = - 0.359  **p = 0.047** | r = 0.548  **p = 0.017** |  |  |  |  |  |
| SSD-n | r = 0.626  **p = 0.037** | r = -0.333  **p = 0.036** | r = 0.485  **p = 0.025** | r = - 0.290  p = 0.059 | r = - 0.535  **p = 0.003** | r = - 0.277  p = 0.081 | r = -0.432  **p = 0.004** | r = - 0.289  p = 0.070 |  |  |  |  |
| BDI | r = - 0.243  p = 0.131 | r = - 0.153  p = 0.347 | r = 0.039  p = 0.810 | r = 0.116  p = 0.478 | r = - 0.107  p = 0.513 | r = - 0.121  p = 0.462 | r = 0.174  p = 0.283 | r = - 0.182  p = 0.260 | r = 0.110  p = 0.506 |  |  |  |
| STAI Trait | r = - 0.332  **p = 0.012** | r = - 0.207  p = 0.206 | r = 0.194  p = 0.237 | r =0.137  p = 0.406 | r = 0.248  p = 0.122 | r = - 0.043  p = 0.793 | r = 0.138  p = 0.415 | r = - 0.289  p = 0.072 | r = 0.077  p = 0.632 | r = 0.662  **p < .001** |  |  |
| STAI State | r = - 0.399  **p = 0.012** | r = - 0.264  p = 0.105 | r = 0.195  p = 0.234 | r = - 0.244  p = 0.135 | r = 0.137  p = 0.400 | r = -0.139  p = 0.412 | r = 0.151  p = 0.352 | r = 0.137  p = 0.400 | r = - =.139  p = 0.386 | r = 0.530  **p < .001** | r =0.760  **p < .001** |  |

**Supplementary Table 1.** Correlations between behavioural measures and control questionnaires assessing depression and anxiety. Results found strong positive associations between levels of state and trait anxiety and depressive symptoms. Higher levels of state and trait anxiety were further associated with shorter reaction times for systole couple distractor trials. However, no association between anxiety and depression emerged for behavioural values assessing inhibitory performance on the stop-signal task.

|  | RT-s | RT-d | RT-n | SSRT-s | SSRT-d | SSRT-n | SSD-s | SSD-d | SSD-n | UPPS NU | UPPS Pre | UPPS Per | UPPS SS |
| --- | --- | --- | --- | --- | --- | --- | --- | --- | --- | --- | --- | --- | --- |
| RT-s |  |  |  |  |  |  |  |  |  |  |  |  |  |
| RT-d | r = 0.314  **p = 0.049** |  |  |  |  |  |  |  |  |  |  |  |  |
| RT-n | r = 0.552  **p = 0.005** | r = - 0.572  **p = 0.039** |  |  |  |  |  |  |  |  |  |  |  |
| SSRT-s | r = - 0.424  **p = 0.006** | r = 0.340  p = 0.054 | r = - 0.342  p = 0.053 |  |  |  |  |  |  |  |  |  |  |
| SSRT-d | r = - 0.371  **p = 0.042** | r = 0.563  **p < .001** | r = 0.449  **p = 0.032** | r = - 0.320  **p = 0.048** |  |  |  |  |  |  |  |  |  |
| SSRT-n | r = 0.755  **p < .001** | r = - 0.439  **p = 0.013** | r = 0.375  p = 0.064 | r = - 0.468  **p = 0.030** | r = - 0.704  **p < .001** |  |  |  |  |  |  |  |  |
| SSD-s | r = - 0.467  **p = 0.002** | r = - 0.645  **p = 0.009** | r = 0.553  **p = 0.003** | r = - 0.374  **p = 0.036** | r = 0. 610  **p = 0.004** | r = -0.230  p = 0.053 |  |  |  |  |  |  |  |
| SSD-d | r = - 0.438  **p = 0.038** | r = -0.402  **p = 0.031** | r = - 0.621  **p = 0.003** | r = 0.514  **p = 0.018** | r = - 0.307  p = 0.058 | r = - 0.359  **p = 0.047** | r = 0.548  **p = 0.017** |  |  |  |  |  |  |
| SSD-n | r = 0.626  **p = 0.037** | r = -0.333  **p = 0.036** | r = 0.485  **p = 0.025** | r = - 0.290  p = 0.059 | r = - 0.535  **p = 0.003** | r = - 0.277  p = 0.081 | r = -0.432  **p = 0.004** | r = - 0.289  p = 0.070 |  |  |  |  |  |
| UPPS NU | r = 0.154  p = 0.341 | r = - 0.194  p = 0.231 | r = 0.132  p = 0.417 | r = -0.017  p = 0.917 | r = 0.025  p = 0.877 | r = 0.032  p = 843 | r = 0.174  p = 0.283 | r = - 0.107  p = 0.513 | r = - 0.043  p = 0.793 |  |  |  |  |
| UPPS Pre | r = - 0.116  p = 0.477 | r = 0.260  p = 0.105 | r = - 0.238  p = 0.139 | r = - 0.007  p = 0.966 | r = 0.057  p = 0.729 | r = - 0.235  p = 0.145 | r = 0.033  p = 0.838 | r = 0.248  p = 0.122 | r = 0.133  p = 0.414 | r = - 0.599  **p < .001** |  |  |  |
| UPPS Per | r = - 0.086  p = 0.598 | r = 0.210  p = 0.194 | r = - 0.189  p = 0.243 | r = 0.174  p = 0.283 | r = 0.202  p = 0.211 | r = - 0.129  p = 0.427 | r = 0.035  p = 0.831 | r = 0.065  p = 0.691 | r = - 0.182  p = 0.260 | r = - 0.575  **p < .001** | r = 0.628  **p < .001** |  |  |
| UPPS SS | r = - 0.214  p = 0.186 | r = 0.136  p = 0.402 | r = - 0.173  p = 0.285 | r = 0.138  p = 0.397 | r = - 0.132  p = 0.418 | r = - 0.276  p = 0.084 | r =0.031  p= 0.849 | r =0.041  p= 0.801 | r = - 0.289  p = 0.070 | r =- 0.475  **p = 0.002** | r = 0.326  **p = 0.037** | r = 0.389  **p = 0.012** |  |

**Supplementary Table 2.** Correlations between behavioural measures and measures of impulsivity (negative urgency, premeditation, perseverance, sensation seeking). Neither reaction times nor measures of inhibitory control significantly correlated with any measures of impulsivity.


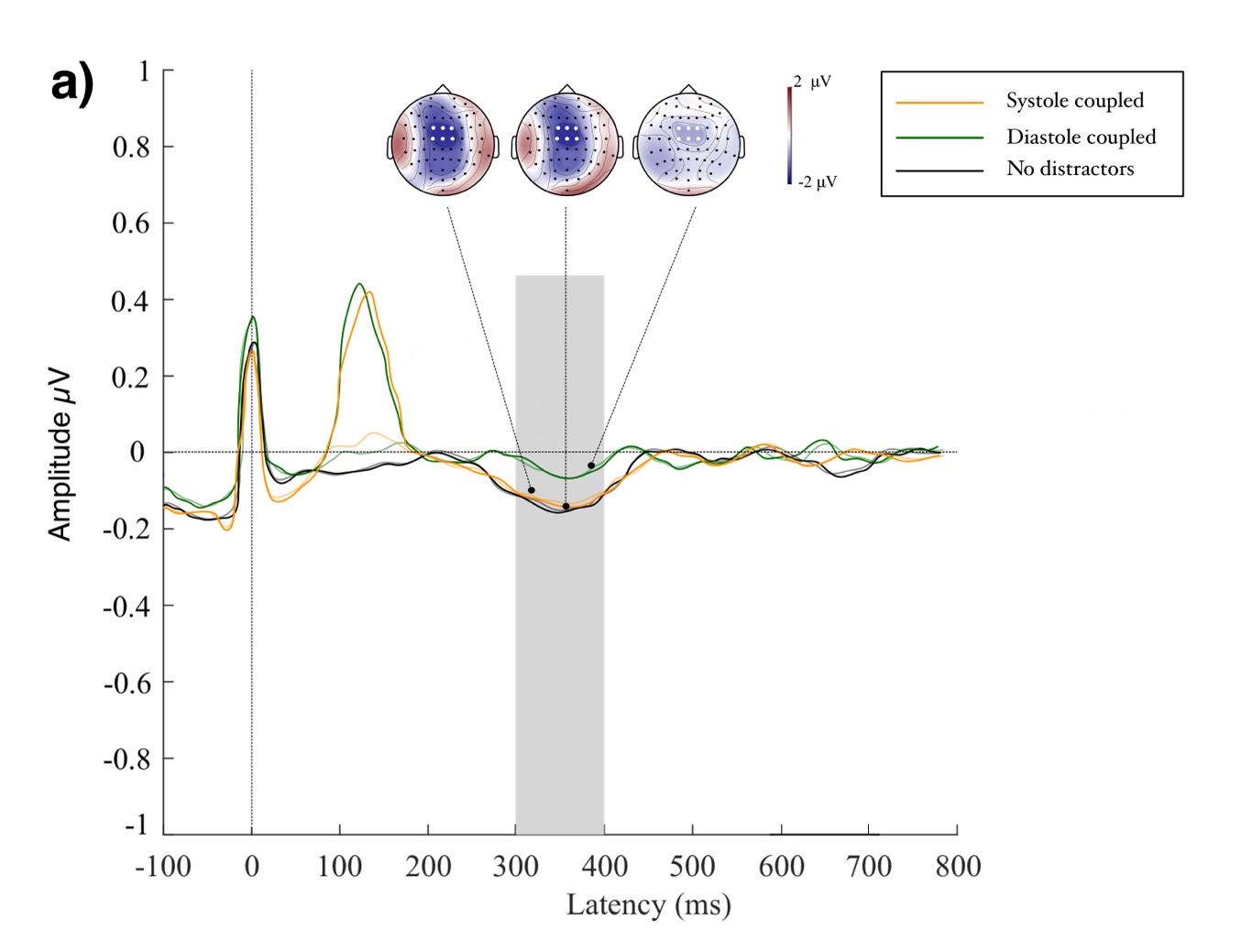


**Supplementary Figure 1.** Grand average waveforms of HEP activity across the three distractor movement conditions. The opaque lines represent HEP amplitudes uncorrected for VEP activity. The lighter lines show HEP waves corrected for VEPs. While early activity (100 to 200 ms) related to visual processing is erased in the corrected dataset, no differences appear in the time window (300 to 400 ms) during which the experimental effect is reflected in HEP activity.

***Control Analysis Heartbeat processing during distractor movement***

To ascertain whether the presence of VEP activity in our HEP dataset affected results reported in the primary analysis, we re-ran our ANOVA on waveforms corrected for VEP amplitudes. The model across the central parietal collection of electrodes returned the same main effect of distractor type [F(2,78) = 7.19, p = 0.001, 𝜂²_p_ =0.04, BF = 4.3]. Post-hoc tests confirmed that HEP activity was significantly reduced in the diastole distractor condition relative to the systole (mean^diff^ = -0.86 µV; p = 0.009, 95% CI [- 0.26, - 0.029, Cohen’s d = 0.35]) and no distractor (mean^diff^ = 0.73 µV, p = 0.003, 95% CI[0.05, 0.27, Cohen’s d = 0.4] ) conditions. We can thus rule out that HEP results were impacted by the overlap of VEP activity.


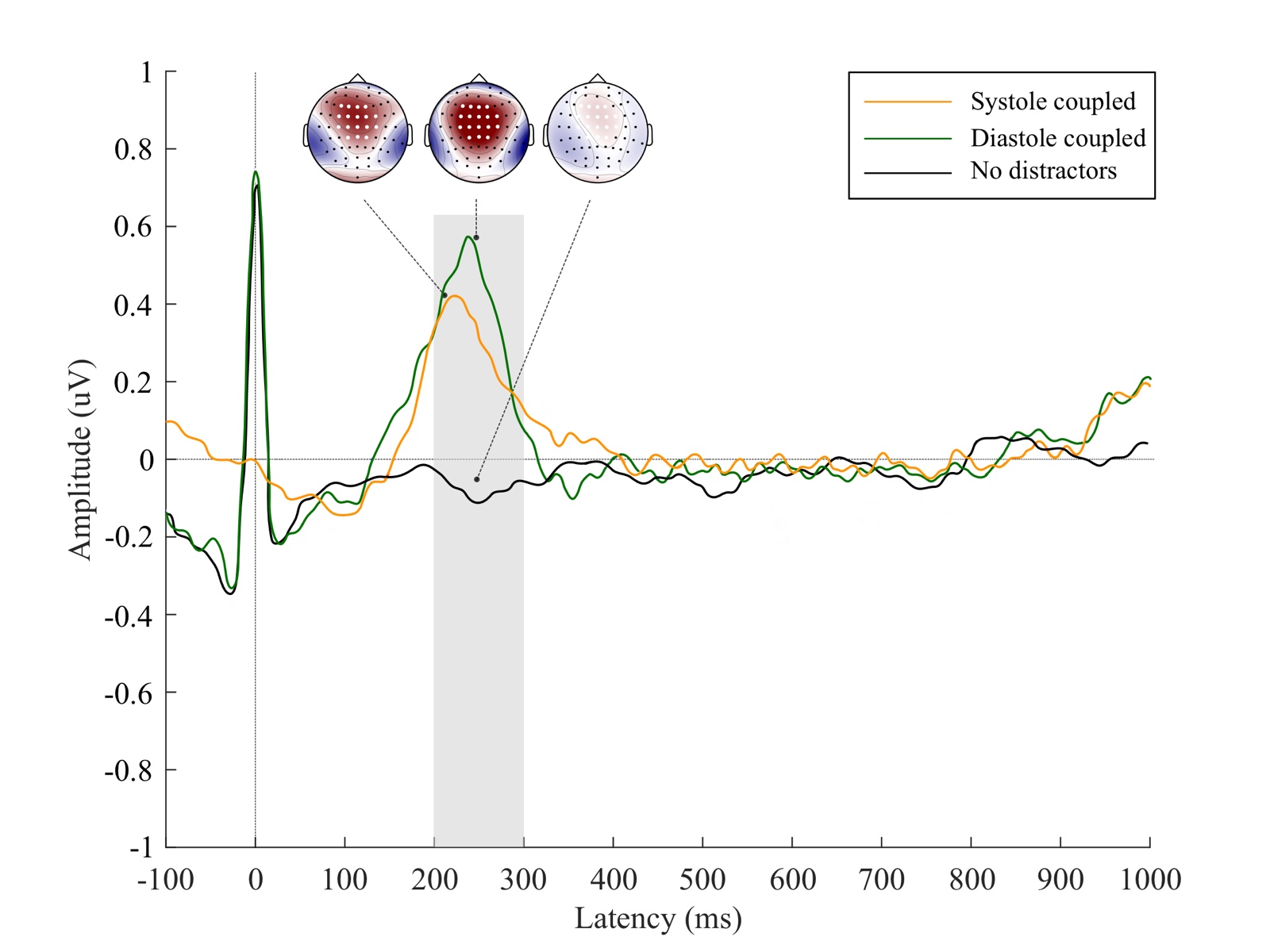


**Supplementary Figure 2a.** Grand average waveforms of VEP activity across the systole, diastole and no distractor movement condition. Without correction, the cardiac field artefact is clearly visible for diastole and no distractor conditions (segmented at R-peak + 0 ms) while it is no longer present for the systole condition (segmented at R-peak + 290 ms).


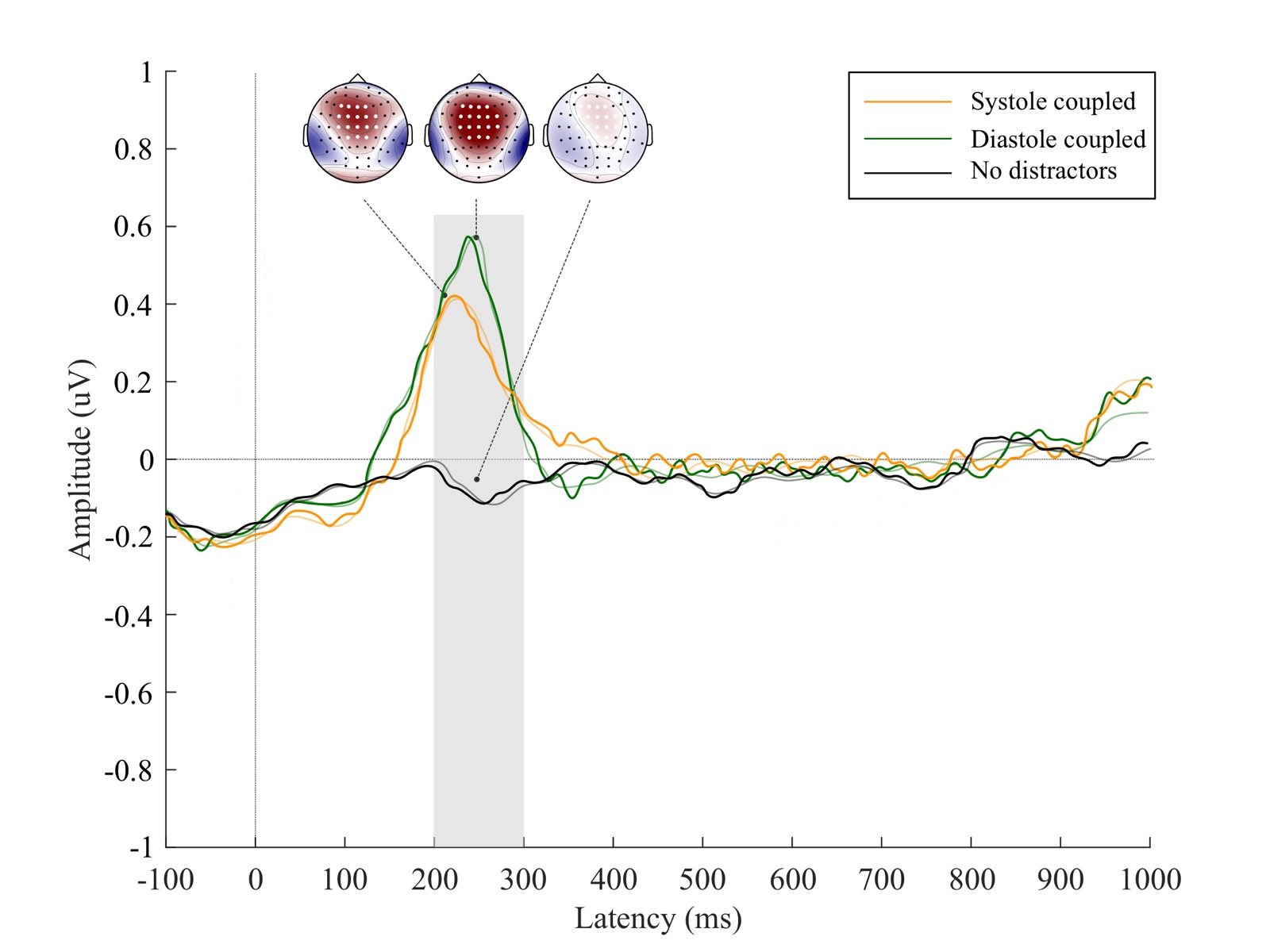


**Supplementary Figure 2b.** Grand average waveforms of VEP activity across the three distractor movement conditions. The opaque lines represent VEP amplitudes uncorrected for HEP activity. The lighter lines show VEP waves corrected for HEPs. Within the time window of interest (200 to 300 ms) for which we report the experimental effect corrected waveforms show hardly any departure from uncorrected ones.

**Control Analysis visual processing of distractor movement**

To ensure the removal of the cardiac field artefact from the VEP data did not lead to spurious effects of our primary analysis, we re-ran the ANOVA on the fronto-central region of electrodes uncorrected for removal of the CFA artefact (Figure 4a). Results found the same main effect of distractor condition reported in the primary analysis [F(1,39) = 12.41, p = 0.001, 𝜂²_p_ =0.57, BF = 10.4]. Post-hoc tests likewise indicated higher VEP amplitude in response to processing the diastole linked distractor movement relative to systole linked distractor movement (mean^diff^ = 0.16 µV, p = 0.001, 95% CI[0.14, 0.89, Cohen’s d = 0.32). We can therefore conclude that removal of the CFA did not impact on findings reported in the primary analysis.

To further ascertain whether the presence of HEP activity may have impacted our VEP findings, we re-ran the ANOVA once again on the same fronto-central cluster of electrodes corrected for potentially infringing HEP activity. Results returned the same main effect of distractor type as the primary analysis uncorrected for HEP activity [F(1,39) = 12.21, p = 0.001, 𝜂²_p_ =0.57, BF = 10.4]. Post-hoc tests confirmed that VEP amplitude was significantly higher for diastole distractor processing compared to the processing of systole distractors (mean^diff^ = 0.15 µV, p = 0.001, 95% CI[0.14, 0.89, Cohen’s d = 0.32). We can therefore conclude that VEP findings reported in the primary analysis were not impacted by HEP activity.

**The relationship between different ERP components**

As a final supplementary analysis of interest , we determined the relationship between the different ERP components. Following a previous procedure^35^, we sorted single trials according to mean ERP amplitude. We then created three equal bins corresponding to low, middle and high ERP amplitude. We created these bins for the HEP, visual evoked activity in response to distractors, motor evoked activity in response to the stop signal and evoked activity in response to the feedback. We then once again applied our cluster-based permutation test across the time window and region of interest our initial permutation test determined to explore whether evoked neural activity differed according to whether foregone ERP activity was high or low. Specifically, we investigated whether motor and feedback evoked activity differed as a function of low or high visual evoked activity and whether feedback activity differed as a function of low or high HEP amplitudes.

**Results**

To test whether different levels of visual P2 activation affected motor (N2) and feedback (P3) activity, we applied a cluster-based permutation t test in the time window of 0 to 600 ms comparing N2 activity and P3 activity following low and high P2 amplitudes. For N2 activity, we observed lower activation across the fronto-central region when stimulus encounter was preceded by high compared to low P2 amplitudes (Monte Carlo P = 0.008). We found no differences in feedback P3 activity succeeding high or low levels of visual P2 activity (Monte Carlo P = 0.07). Results hereby show a direct association between visual and motor cortical activity, suggesting that greater suppression of cortical visual activity towards distractors has downstream implications for improved motor activation.

To investigate the association between HEP amplitude and P2 visual evoked activity, we ran a further permutation test in the time window of 0 to 600 ms, exploring P2 activity following high vs. low amplitudes of the HEP. We observed lower P2 amplitudes across the fronto-central region when encounter of the visual distractors coincided with high levels of HEP activity (Monte Carlo P = 0.006). Results hereby suggest that presenting stimuli in synchrony to cortical heartbeat feedback influences interoceptive cardiac processing as well as the ability to successfully suppress visual cortical activity.
